# The Association of Antipsychotic Treatment and Side Effects With Societal Recovery and Happiness: A Naturalistic Cohort Study of People in Long-term Care for a Psychotic Disorder

**DOI:** 10.1093/schbul/sbaf122

**Published:** 2025-08-13

**Authors:** Stijn Crutzen, Shiral Gangadin, Ken Ho Hua, Ellen Visser, Frederike Jörg, Gerdina Hendrika Maria Pijnenborg, Lisette van der Meer, Wim Veling, Stynke Castelein

**Affiliations:** Rob Giel Research Center, University Center Psychiatry, University Medical Center, University of Groningen, Hanzeplein 1, Groningen P.O. 30.001, 9700 RB, the Netherlands; Lentis Research, Lentis Psychiatric Institute, Hereweg 80, Groningen 9725 AG, the Netherlands; Rob Giel Research Center, University Center Psychiatry, University Medical Center, University of Groningen, Hanzeplein 1, Groningen P.O. 30.001, 9700 RB, the Netherlands; Apotheek Spaarne Gasthuis, Spaarne Gasthuis, Haarlem/Hoofddorp P.O. 417, 2000 AK, the Netherlands; Rob Giel Research Center, University Center Psychiatry, University Medical Center, University of Groningen, Hanzeplein 1, Groningen P.O. 30.001, 9700 RB, the Netherlands; Rob Giel Research Center, University Center Psychiatry, University Medical Center, University of Groningen, Hanzeplein 1, Groningen P.O. 30.001, 9700 RB, the Netherlands; Faculty of Behavioural and Social Sciences, University of Groningen, Grote Kruisstraat 2/1, Groningen 9712 TS, the Netherlands; Department of Psychotic Disorders, GGZ Drenthe Mental Health Institution, Dennenweg 9, Assen 9404 LA, the Netherlands; Department of Clinical and Developmental Neuropsychology, University of Groningen, Groningen 9712 TS, the Netherlands; Department of Rehabilitation, Lentis Psychiatric Institute, Zuidlaren 9470 AC, the Netherlands; Psychosis Department, University Center for Psychiatry, University Medical Center Groningen, University of Groningen, Groningen, the Netherlands; Lentis Research, Lentis Psychiatric Institute, Hereweg 80, Groningen 9725 AG, the Netherlands; Faculty of Behavioural and Social Sciences, University of Groningen, Grote Kruisstraat 2/1, Groningen 9712 TS, the Netherlands

**Keywords:** schizophrenia, social functioning, well-being, quality of life

## Abstract

**Background:**

Antipsychotics are used to manage symptoms and reduce the risk of relapse. However, the antipsychotic side effects are associated with a lower quality of life and are seen as major barriers to achieving societal recovery by antipsychotic users. In this study, we investigate the association of side effects, antipsychotic dose, and antipsychotic polypharmacy with societal recovery and happiness.

**Study Design:**

Data were used from a large, naturalistic, longitudinal cohort of people using an antipsychotic in long-term care (Pharmacotherapy Monitoring and Outcome Survey [PHAMOUS], 2013-2021). The association between subjective antipsychotic side-effect burden (measured with the Subjective Response to Antipsychotics questionnaire), antipsychotic dose, and antipsychotic polypharmacy with societal recovery and happiness was investigated using mixed-effect linear regression models. In an exploratory analysis, the associations between individual side effects with societal recovery and happiness were assessed.

**Study Results:**

Data from 5971 observations nested in 2490 participants were used. The subjective antipsychotic side-effect burden, total antipsychotic dose, and antipsychotic polypharmacy were significantly negatively associated with societal recovery. Subjective antipsychotic side-effect burden and total antipsychotic dose were significantly negatively associated with happiness, but antipsychotic polypharmacy was not. Cognitive, mood, and physical anticholinergic side effects were most strongly negatively associated with societal recovery. Mood-, sedation-, cognitive-, and sexual-related side effects were most strongly negatively associated with happiness.

**Conclusions:**

These results show that side effects and a higher dose of antipsychotic medication are negatively associated with societal functioning and happiness. Future research should focus on whether dose reduction is beneficial for societal recovery and happiness in the long-term.

## Introduction

In people with a psychotic disorder, antipsychotics are used to manage positive symptoms and reduce the risk of psychotic relapse. About half of patients reach clinical recovery after a first-episode psychosis (FEP), which is partially due to treatment with antipsychotics. ^1^^,^^2^ However, full recovery is often not achieved for many people with a psychotic disorder.^1–5^ Concepts used to describe and investigate full recovery from a first psychotic episode have been diverse and often overlap; however, 3 core components of recovery can be distinguished. Besides clinical recovery, regaining daily functioning and promoting wellbeing are essential in achieving full recovery. Concepts related to daily functioning used are social functioning, occupational functioning, functional recovery/remission, and societal recovery (i.e., regaining everyday functioning in work, housing and social relations^6^). Closely overlapping concepts often used in research to assess wellbeing are quality of life and happiness (*subjective evaluation of quality of life*^7^^,^^8^), health-related quality of life, and personal recovery (i.e., living a meaningful life^9^).^10^

In contrast to clinical recovery, societal recovery and quality of life show less improvement over the years.^4^^,^^5^ A recent meta-analysis revealed that in the long term only about one-third of patients were employed and only around 20% were in a relationship.^4^ Another meta-analysis found only small to moderate improvements in personal recovery and subjective quality of life among people with schizophrenia.^5^ Which type of recovery patients want to prioritize in their treatment highly varies between patients.^11^^,^^12^ While some prioritize clinical recovery, many rate treatment goals related to personal and societal recovery as more important.^11–14^

While antipsychotic treatment can contribute to reaching clinical recovery, the side effects associated with antipsychotic treatment are seen as major barriers to achieving societal recovery and they are reported to negatively affect quality of life. Most side effects have been shown to be dose dependent and antipsychotic polypharmacy (the use of 2 or more antipsychotics) has been associated with a lower tolerability.^15–19^ Qualitative research shows that many antipsychotic users consider antipsychotics essential to navigate the chaos during the acute phase of psychosis; however, in the long term, they often emphasize the detrimental and debilitating results from the side effects of antipsychotics.^20–23^ Antipsychotic users link these side effects to difficulties with fully engaging in life and social roles, as well as being unable to find employment.^20^^,^^23–25^ Antipsychotic users link in particular side effects like fatigue, sedation, and cognitive problems to impaired daily functioning and the inability to work, impairing societal recovery. Quantitative studies show that antipsychotic side effects were linked to a lower quality of life and lower functioning.^26^^,^^27^ Specifically, side effects such as sedation, cognitive problems, weight gain, and sexual dysfunction were linked to lower health-related quality of life in people with schizophrenia.^27^ Moreover, highly visible side effects like extrapyramidal side effects and weight gain can increase stigma and reduce self-esteem, which in turn can lead to social withdrawal.^20^^,^^23^^,^^28^^,^^29^

Evidence from randomized controlled trials (RCTs) largely contradicts experiences from antipsychotic users. Randomized controlled trials show overall beneficial effects of antipsychotic treatment on functioning and quality of life, both in trials comparing antipsychotic treatment to placebo as well as trials comparing antipsychotic maintenance to discontinuation.^30^^,^^31^ However, due to short follow-ups, these RCTs fail to elucidate the long-term effects of antipsychotic treatment. Benefits for functioning and quality of life are likely a result of the acute effects of antipsychotics on alleviating positive symptoms and reducing the risk of relapse. As indicated by antipsychotic users, over time, the balance of costs and benefits might tip toward higher burden, as initial gains in symptom reduction give way to more burden from side effects.^23^ One of the sparse discontinuation RCTs with an extended follow-up illustrated the potential long-term detrimental effects of antipsychotic treatment.^32^ After 7 years, functional remission (a concept closely related to societal recovery as it was defined as minimal disability in the last 6 months in self-care, housekeeping, family relationships, partner relationships, relationships with peers, community integration, and vocational functioning) was achieved by approximately twice as many patients in the reduction/discontinuation group, while there were no differences in clinical recovery. In contrast, the only other long-term discontinuation RCT showed poorer clinical outcomes and no benefits in quality of life and social and occupational functioning (2 out of 3 components of societal recovery).^33^

When evaluating antipsychotic treatment, it is important to examine beyond the risk of psychosis relapse, psychotic symptoms, and side effects in the short-term, and to include the long-term effects of antipsychotic treatment on societal recovery and quality of life/happiness in this equation. Current evidence from RCTs leave us with 2 major gaps in knowledge. Randomized controlled trials fail to disentangle the positive effects (symptom reduction and reduction in relapse rate) from the negative effects (side effects) on societal functioning and quality of life/happiness and they fail to show the effects of antipsychotic treatment in the long-term. Therefore, in this study, we investigate the association of side effects, antipsychotic dose, and antipsychotic polypharmacy with societal recovery and happiness in a population of antipsychotic users in long-term care.

## Methods

### Design and Participants

Data from the Pharmacotherapy Monitoring and Outcome Survey (PHAMOUS) were used, a large naturalistic longitudinal cohort of people using an antipsychotic in long-term care.^34^ As an annual routine outcome monitoring, PHAMOUS is used for the evaluation of treatment of individuals with a psychotic disorder as well as to study treatment on a population level. Data were gathered from 4 mental health institutions in the northern parts of the Netherlands. This cohort has been described in more detail in a previous publication.^34^ Based on the availability of the Subject’s Response to Antipsychotics (SRA-34), which assesses subjective side-effect burden of antipsychotics, we used data from 2013 to 2021. In this study, patients were included if they completed the subjective side-effects burden (SRA-34) questionnaire, were 18 years or older, met the criteria for a psychotic disorder based on the DSM-IV/DSM-5,^35^^,^^36^ used an antipsychotic at the time of the measurement, based on the level 3 antipsychotics group (N05A) excluding lithium (N05AN01) and phenothiazines with aliphatic side-chain (N05AA) from the Anatomical Therapeutic Chemical (ATC) Classification System and when the total antipsychotic dose was available.

### Outcomes

#### Societal Recovery and Happiness

Societal recovery was assessed with the Functional Recovery tool (FRt).^37^^,^^38^ The term societal recovery was used instead of functional recovery to make the distinction between functional recovery as defined as recovery from cognitive and mental impairment or being able to function with these impairments,^6^ this was done in line with previous research conducted with the PHAMOUS cohort.^9^^,^^39^^,^^40^ The FRt was scored by a clinician on 3 domains: (1) daily living and self-care, (2) work, study and housekeeping, and (3) social contacts. The 3 domains were scored from “independent” (score 0), “partially independent” (score 1) to “not independent” (score 2). Zero represents normal functioning, which was defined as an absence of any problem or need for help in this domain. When an item is scored with one, a clear lack of proficiency in this domain is present, for which support is needed. Finally, a score of 2 represents a complete inability to function in the domain. The reversed total sum score (0-6) was used to improve interpretability with a higher score indicating a higher level of societal recovery. The FRt showed adequate internal reliability and acceptable test–retest reliability. Modest convergent validity was shown with the Camberwell Assessment of Need Short Appraisal Schedule domains: daily living, work, and social contacts. Most research on antipsychotics focuses on quality of life. The current study uses happiness (i.e., *subjective evaluation of quality of life*^7^^,^^8^*)* as outcome, which has demonstrated to be strongly correlated with quality of life.^10^ Happiness was used instead of quality of life because some of the items in the quality-of-life questionnaire administered as part of the PHAMOUS screening, namely, the Manchester Short Assessment of Quality of Life (ManSA),^41^^,^^42^ show overlap with societal and clinical recovery. For example, some items in this questionnaire are about satisfaction with employment, income, relationships, and mental health. Happiness was measured with the Single-Item Happiness Question (SIQ), which is a self-reported questionnaire and scored on a scale from 0 to 10.^7^ The SIQ showed good test–retest reliability.^7^ Concurrent and convergent validity was demonstrated through correlations with the Oxford Happiness Inventory and the Satisfaction with Life Scale and with optimism, hope, self-esteem, positive affect, extraversion, physical health, and mental health.

#### Antipsychotic Polypharmacy and Dose

Patients were asked to bring medication prescriptions to the assessment, and the medication was registered by trained research nurses.^34^ Antipsychotic polypharmacy was defined as the simultaneous use of 2 or more antipsychotics (ATC N05A), excluding lithium (N05AN01) and phenothiazines with aliphatic side-chain (N05AA). The risperidone equivalent dose was calculated based on the methods used recommended in the meta-analysis by Leucht et al. (2021).^19^

#### Subjective Antipsychotic Side-Effect Burden

The subjective antipsychotic side-effect burden was measured with the SRA-34.^43^ The SRA-34 contains 10 items about desired effects of antipsychotics and 24 items about undesired effects of antipsychotics. For this article, the items about the undesired effects were used. In these items, antipsychotic users were asked whether they experienced a certain undesired effect (side effect) due to their antipsychotics which they could rate on a scale from not present (0), yes, mild (1) to yes, severe (2). A total score for the unwanted effects was calculated by adding all 24 unwanted items. Missing items were considered not present (0). When more than 4 items were missing, the patient was excluded from analysis.

#### Data Analysis

In the PHAMOUS screening, patients can receive multiple measurements over the years. PHAMOUS is set up as an annual screening. However, intervals between measures and the total number of observations per patient can vary highly. Patient-level characteristics were reported for the descriptive analyses, because observations are nested in patients. For continuous variables, a weighted mean was calculated, and for categorical variables, the mode (i.e., the most prevalent outcome over all observations per individual) was used for the descriptive statistics. For the inferential statistics, mixed-effects linear regression models were used to investigate the association between antipsychotic side effects, antipsychotics dose, and antipsychotic polypharmacy with societal recovery and happiness. Mixed-effect models were used to account for the nesting of observations in patients. A random intercept and a random slope for calendar year were used in all models. In sensitivity analyses, age, sex, duration of illness, and positive symptoms as measured by the Positive and Negative Syndrome Scale (PANSS)^44^ were added to the mixed-effect models. The variance inflation factor (VIF) was used to check for collinearity. Duration of illness and positive symptoms were adjusted for because of their association with more antipsychotic treatment and societal recovery.^45^^,^^46^ We did not include negative and general symptoms as covariates, because some antipsychotic side effects overlap with some of the negative and general symptoms, which can make it hard to make the distinction between side effects and symptoms.^47^ In an exploratory analysis, mixed-effect linear regression models were used to investigate the association of the 24 individual subjective side effects with societal recovery and happiness. For each individual side effect, a mixed-effect linear regression model was used. The items were dummy coded with no side effect as the reference value. In addition, in a subgroup analysis, data were split based on sex.

#### Ethics

Pharmacotherapy Monitoring and Outcome Survey is an ongoing routine outcome monitoring program and naturalistic Dutch cohort study that started in 2006. It runs in 4 mental health institutions in the northern Netherlands and is part of regular care. All people with psychotic disorders and/or people using antipsychotic medication are invited to participate in annual screenings, with no exclusion criteria for participation. During the screening, trained nurses examine participants’ mental and physical health. A report is made and used by service users and practitioners to evaluate or set up their treatment plan. According to the declaration of Helsinki, service users are informed that their pseudonymized data could be used for scientific research, and they have the option to opt out of data use. Research on this dataset has been deemed exempt from the Medical Research Involving Human Subjects Act (WMO) by the Medical Ethical Committee of the University Medical Center Groningen. This exemption was granted because no additional burden is placed on service users, the research involves pre-existing routine healthcare data, and research on the data serves a broad public interest.

## Results

In total, 5971 observations nested in 2490 patients were used. About one-third were female, on average, participants were 44.1 years old and about half (52.5%) had more observations in symptomatic remission than not. The majority of patients (68.1%) were diagnosed with schizophrenia and the average duration of illness was 17.4 years (Table 1).

**Table 1:** Patient-Level Characteristics

Number of patients		2490
Number of observations per patient, mean (range)		2.4 (1–9)
Time between observations (years), mean (SD)		1.5 (1.1)
Age (years), mean (SD)		44.1 (11.7)
Sex (female), %		33.8%
Happiness (0–10), mean (SD)		6.5 (1.6)
Societal recovery (0–6), mean (SD)		3.4 (1.6)
Societal remission,^a^ mode %		26.5%
Symptomatic remission,^b^ mode %		52.5%
PANSS positive symptom subscale, mean (SD)		11.6 (4.3)
PANSS negative symptom subscale, mean (SD)		13.1 (5.4)
PANSS general psychopathology subscale, mean (SD)		25.6 (7.3)
Duration of illness, mean (SD)		17.4 (11.6)
Total subjective antipsychotic side-effect burden, mean (SD)		10.9 (7.2)
Total risperidone equivalent dose (mg), mean (SD)		5.5 (5.0)
Antipsychotic polypharmacy, mode %		22.2%
Main diagnosis, mode %		
Schizophrenia		68.1%
Delusional disorder		2.2%
Schizophreniform disorder		0.9%
Substance abuse psychosis		1.0%
Schizoaffective disorder		3.4%
Psychosis NOS		13.8%
Unknown psychotic disorder		10.7%

Abbreviations: NOS = not otherwise specified; PANSS = Positive and Negative Syndrome Scale; SD = standard deviation

a A score of 5 or 6 on the Functional Recovery tool (FRt) is considered societal remission.

b A score of 3 or lower on 8 items from the PANSS defined by The Remission in Schizophrenia Working Group.^48^

### Societal Recovery

Observations with a missing score for the FRt were excluded, which resulted in a total of 4924 observations nested in 2236 patients that were used for the analyses. The VIF was low (<1.7) for all covariates. An intraclass correlation of 0.663 was found indicating that level of societal recovery was relatively stable over the years. Table 2 shows that the total subjective side-effect burden, total antipsychotic dose, and antipsychotic polypharmacy were statistically significantly negatively associated with societal recovery. In the sensitivity analysis, a total of 3815 observations nested in 1809 patients were included. The sensitivity analysis showed that when correcting for sex, age, positive symptoms, and duration of illness these associations remained statistically significant. A longer duration of illness, positive symptoms, and being male were all associated with a lower level of societal recovery.

In Table A1 of Appendix 1, the results of the association of societal recovery with the 24 individual side-effect items are shown. Subjective side effects that had a relatively strong association with societal recovery were cognition-related side effect (diminished creativity, slower reaction, and difficulty remembering), mood (depression, less energy for social interactions, and blunted affect), sedation-related side effects (less energy for social interactions, depression, and physical tiredness), muscle stiffness, urine loss, and physical anticholinergic side effects (constipation, blurry vision, and dry mouth). The subgroup analysis (Table A3 of Appendix 1) shows that physical tiredness was only associated with societal recovery for men.

### Happiness

In the mixed-effect linear regression model, after excluding observations without a happiness score, a total of 5574 observations nested in 2423 patients were included. The VIF was low (<1.7) for all covariates. An intraclass correlation of 0.483 was found indicating that happiness was relatively stable over the years. Table 3 shows that total subjective side-effect burden and total antipsychotic dose were statistically significantly negatively associated with happiness, while antipsychotic polypharmacy was not. In the sensitivity analyses, a total of 4256 observations were used nested in 1959 patients. The sensitivity analysis showed that when correcting for sex, age, positive symptoms, and duration of illness, subjective total side-effect burden remained statistically significantly associated with happiness, while the association with the total antipsychotic dose was not statistically significant. A shorter duration of illness, positive symptoms, and being male were associated with lower happiness.

In Table A2 of Appendix 1, the results of the association of happiness with the 24 individual side-effects item are shown. Subjective side effects that had a relatively strong association with happiness were mood-related side effects, cognition-related side effects, sedation-related side effects, muscle stiffness, and sexual side effects (lower libido and difficulties reaching orgasm). Subgroup analysis (Table A4, Appendix 1) showed that lower libido had a stronger association with happiness for men than for women. Increased appetite and weight gain showed a small association with happiness for women, while for men these side effects were not associated with a lower happiness. Difficulties sitting still (akathisia) was only associated with happiness for men.

**Table 2:** Results of Mixed-Effect Linear Regression Model Showing the Association of Subjective Side-Effect Burden, Total Antipsychotic Dose and Antipsychotic Polypharmacy With Societal Recovery

	Beta coefficient	Standard error	z-score	*P*-value	95% CI	
					Lower bound	Upper bound
**Main analysis**						
Total subjective antipsychotic side-effect burden	−0.0182	0.0032	−5.72	**<.001**	−0.0244	−0.0119
Total risperidone equivalent dose (mg)	−0.0286	0.0053	−5.40	**<.001**	−0.0389	−0.0182
Antipsychotic polypharmacy	−0.244	0.0593	−4.11	**<.001**	−0.360	−0.128
Time (years)	0.0067	0.0083	0.80	.423	−0.0096	0.0230
**Sensitivity analysis**						
Total subjective antipsychotic side-seffect burden	−0.0137	0.0036	−3.78	**<.001**	−0.0207	−0.0066
Total risperidone equivalent dose (mg)	−0.0178	0.0059	−3.00	**.003**	−0.0389	−0.0182
Antipsychotic polypharmacy	−0.234	0.0659	−3.55	**<.001**	−0.363	−0.105
Female sex	0.519	0.0722	7.19	**<.001**	0.378	0.661
Age (years)	−0.0073	0.0038	−1.93	.053	−0.0148	0.00009
Positive symptoms	−0.0756	0.0058	−12.93	**<.001**	−0.0870	−0.0641
Duration of illness (years)	−0.0141	0.0039	−3.64	**<.001**	−0.0217	−0.0065
Time (years)	0.0183	0.0096	1.91	.056	−0.0005	0.0371

P-values ≤0.05 are statistically significant

**Table 3:** Results of Mixed-Effect Linear Regression Model Showing the Association of Subjective Side-Effect Burden, Total Antipsychotic Dose, and Antipsychotic Polypharmacy With Happiness

	Beta coefficient	Standard error	z-score	*P*-value	95% CI	
					Lower bound	Upper bound
**Main analysis**						
Total subjective antipsychotic side-effect burden	−0.0571	0.0032	−17.74	**<.001**	−0.0635	−0.0509
Total risperidone equivalent dose (mg)	−0.0132	0.0053	−2.49	**.013**	−0.0235	−0.0028
Antipsychotic polypharmacy	−0.0437	0.0612	0.71	.475	−0.0762	0.164
Time (years)	0.0189	0.0088	2.15	**.032**	0.0017	0.0362
**Sensitivity analysis**						
Total subjective antipsychotic side-effect burden	−0.0489	0.0036	−13.46	**<.001**	−0.0560	−0.0418
Total risperidone equivalent dose (mg)	−0.0109	0.0057	−1.91	.056	−0.0221	0.0003
Antipsychotic polypharmacy	0.0666	0.0670	0.99	.320	−0.0647	0.198
Female sex	0.163	0.0681	2.39	**.017**	0.0293	0.296
Age (years)	−0.0014	0.0036	−0.41	.680	−0.0085	0.0055
Positive symptoms	−0.0679	0.0058	−11.62	**<.001**	−0.0870	−0.0641
Duration of illness (years)	0.0122	0.0037	3.32	**.001**	0.0050	0.0193
Time (years)	0.0179	0.0100	1.79	.073	−0.0017	0.0373

P-values ≤0.05 are statistically significant

## Discussion

This study investigated the association of antipsychotic treatment and its side effects with societal recovery and happiness in a population of patients with a psychotic disorder who are in long-term care and who experienced on average mild psychotic symptoms. The subjective antipsychotic side-effect burden, total antipsychotic dose, and antipsychotic polypharmacy were negatively associated with societal recovery. These associations remained statistically significant after correcting for sex, age, positive symptoms, and duration of illness. Subjective antipsychotic side-effect burden and total antipsychotic dose were negatively associated with happiness, no association was found for happiness with antipsychotic polypharmacy. After correcting, the association between subjective side-effect burden with happiness remained significant. Overall, the associations were relatively small. On average 10 more severe side effects were associated with a 0.36-point lower score on the FRt (score ranged from 0 to 6). The association with happiness was larger. On a scale from 0 to 10, 10 more severe side effects were associated with a 1.1-point lower score. The associations with individual side effects were larger. Cognition, mood, and physical anticholinergic side effects were most strongly associated with societal recovery. Mood-, sedation-, cognitive-, and sexual-related side effects were most strongly associated with happiness.

### Comparison with Literature

#### Societal Recovery

Relatively little research has been done, which focused on the relationship of societal recovery with antipsychotic treatment in patients in long-term care. A recent meta-analysis showed that maintained antipsychotic treatment in the short term was associated with better social functioning, however, in the middle (2-5 years) to long term (>5 years), discontinuation/dose reduction was associated with higher social functioning.^49^ While short-term RCTs favored maintenance, this effect reduced with longer follow-up.^49^ In line with our results, this suggests that long-term antipsychotic treatment may hinder societal/social recovery. However, the results of this meta-analysis are tentative. The results for the middle- and long-term were based on nonrandomized studies, overall risk of bias was high, and the number of studies was limited. A Danish nationwide cohort study showed that attempting to discontinue antipsychotics was associated with a higher chance of employment and a higher risk of involuntary psychiatric hospitalization,^50^ indicating that there might be a trade-off between symptomatic stability and societal recovery. Still, previously mentioned studies have a high risk of bias by indication. Antipsychotic users who are functioning better might more often initiate and persist with dose reductions or discontinuation.

The side effects of antipsychotic treatment are proposed as the main reason for lower societal recovery.^20^^,^^23^^,^^24^ However, our analysis showed that antipsychotic dose and antipsychotic polypharmacy were associated with societal recovery, independently of subjective side-effect burden. These associations persisted after correcting for positive symptoms and duration of illness, making it less likely that they were caused by a bias by indication. Still, some bias by indication might persist. For some patients, antipsychotic polypharmacy or a high antipsychotic dose^51^ may indicate severe symptoms and relapses in the past. These past symptoms and relapses could have hindered education, stable employment, stable housing, maintaining relationships, and developing new relationships, impacting societal recovery down the line. In other words, relapses in the past and past severe positive symptoms could have influenced current antipsychotic treatment and they might also have influenced current societal recovery and happiness, confounding the association between side effects and antipsychotic treatment with societal recovery. Another explanation for the association of antipsychotic dose and antipsychotic polypharmacy with societal recovery might be that some side effects, like cognitive problems are hard to recognize by antipsychotic users after long antipsychotic treatment.

The side effects that show the strongest association with societal recovery confirm in most cases what is mentioned by antipsychotic users.^20^^,^^23^^,^^24^ The strongest associations were found with side effects related to cognition, mood (e.g., blunted affect and depression), and sedation. Cognitive impairments are well-known predictors of functioning in psychosis.^45^ The way cognitive impairments impact functioning is complex.^52^ The impact of cognitive impairment on functioning is likely mediated and moderated by negative symptoms.^52^ Cognitive impairments can hinder different aspects of societal recovery, for example, poor verbal working memory can reduce social competence, mediated by reduced emotional perspective taking.^53^ Cognitive impairments as a result of the psychotic disorder and some expressive (including blunted affect) and experiential (including anhedonia) negative symptoms overlap with antipsychotic side effects.^47^ Both cognitive impairments and the expressive deficits and experiential domains of negative symptoms can lead to worse psychosocial functioning,^54^ which could impact societal recovery, especially the social domain. Moreover, daytime sleepiness caused by quetiapine has been shown to be associated with worsening of functional capacity.^55^ Sedation in part might impact societal functioning due to a negative effect on motivation.^56^ The concurrence between qualitative and quantitative studies strengthens the hypothesis that these side effects lead to lower societal functioning. Two other types of side effects that showed a strong association in our analysis were muscle stiffness and physical anticholinergic side effects (blurred vision, constipation, and dry mouth). The strong association with physical anticholinergic side effects does not align with the results of qualitative studies.^20^^,^^23^^,^^24^ The physical anticholinergic side effects might directly affect societal recovery (e.g., feeling unsafe to go outside due to blurred vision); however, they might be an indication of a strong anticholinergic effect. Anticholinergic medication is well known for sedation- and cognition-related side effects.^57^ The physical anticholinergic side effects are easier to attribute to antipsychotic treatment than the cognitive anticholinergic side effects, as cognitive problems are often part of the psychotic disorder, which can lead to misclassifying the side effects as symptoms.^47^ A secondary finding was that women showed more societal recovery than men. A recent comprehensive analysis of sex differences in recovery from psychotic disorders using data from the PHAMOUS cohort yielded similar findings. Beyond more recovery in women, this study showed that people with late-onset psychosis showed better outcomes, with women with late-onset psychosis having the best chance of favorable outcomes.^40^

#### Happiness

Previous research showed an association between side effects and health-related quality of life.^26^^,^^27^ Specifically, sedation, cognitive problems, weight gain, and sexual dysfunction were associated with lower health-related quality of life.^27^ Our results confirmed these findings, except for weight gain, which had a relatively small association with happiness for women only. Antipsychotics can have a direct effect on happiness and an indirect effect due to the side effects. Since most antipsychotics are dopamine 2 receptor antagonists, their use can directly influence subjective well-being. Dopamine is well known for its role in the reward system and has been associated with positive affect and with mediating the link between oxytocin and social relationships.^58^^,^^59^ Randomized controlled trials show that in patients treated with antipsychotics, dopamine 2 receptor occupancy shows a strong association with subjective well-being.^60^^,^^61^ Moreover, a non-randomized trial showed that switching to a partial dopamine agonist (aripiprazole) improves subjective well-being.^62^ Antipsychotics can also indirectly affect quality of life/happiness. Indirectly, the negative impact of side effects on societal recovery can decrease happiness/quality of life. Previous research has shown that these forms of recovery are highly connected.^9^ For example, fewer social contacts have been associated with a lower quality of life, possibly due to a higher sense of stigma and lower empowerment, leading to depression.^63^ Employment can improve quality of life by increasing social contacts, improving sense of self-worth and by providing meaning in life.^64^

There is a myriad of ways in which antipsychotic side effects can influence happiness beyond the effects on societal functioning, including highly visible side effects such as weight gain and extrapyramidal side effects, which can increase stigma and self-stigma and can reduce self-esteem, resulting in social withdrawal and lower quality of life.^20^^,^^23^^,^^28^^,^^29^^,^^65^^,^^66^ Blunted affect has been associated with an increased risk of suicide likely mediated by an increase in depressive symptoms, feelings of hopelessness, emotional withdrawal, low self-esteem, and an inability to express affection.^67^ Akathisia and the restlessness associated with akathisia has been associated with depression and suicidality in men.^68^

In contrast to our results, a meta-analysis showed no difference between maintenance and discontinuation/ dose reduction on quality of life.^49^ The included RCTs were all short term (< 2 years). As described by patients that use antipsychotics, antipsychotic use can be a double-edged sword.^23^^,^^69^ While the side effects of antipsychotics might have a negative effect on long-term quality of life, a lack of improvement in quality of life when discontinuation or reducing the dose of antipsychotics could be a result from more relapses in the first 2 years of discontinuation/dose reduction.^70^ Long-term effects of antipsychotic dose reduction and discontinuation are largely unknown.^32^^,^^33^ This includes long-term effects on personal recovery, quality of life, and happiness.

### Strengths and Limitations

The use of a large naturalistic dataset, containing almost 2500 patients and almost 6000 observations, was an important strength. Using a large naturalistic dataset increased ecological validity and enabled a comprehensive examination into a largely underexplored subject. Using observational data, however, does come with some inherent limitations. For instance, the relatively strong association between subjective side effects and happiness could in part be explained by reversed causality or unmeasured confounding: people with more depressive symptoms are known to report more subjective side effects, even side effects that are not caused by antipsychotics (nocebo effect).^71^ Still, similar associations were found for societal recovery, which was rated by a healthcare professional. Moreover, by using a subjective measure for antipsychotic side effects, the personal difficulties perceived by individuals on antipsychotic therapy were reflected more closely. In the sensitivity analyses, we did not correct for negative and general symptoms because some antipsychotic side effects overlap with some of the negative and general symptoms, which can be hard to distinguish.^47^ Part of the associations with the subjective side-effect burden might be due to negative or general symptoms being misinterpreted as side effects or because patients with more negative or general symptoms report more side effects. A single-item questionnaire was used to assess happiness, which may lack comprehensiveness to measure a highly multidimensional, hard to define, and broad in scope concept such as happiness.^72^ Only patients that completed the SRA-34 could be included in the analyses. This could have resulted in a selection bias. Patients for whom side effects were of lesser concern might have completed the SRA-34 less often, which could have resulted in an overestimation of the association of side effects with societal recovery and happiness. Given the long average duration of illness, the results should not be generalized to the FEP population. In fact, from both qualitative and quantitative research, the results are that any potential negative effects of antipsychotic treatment on societal recovery and happiness are to be expected in the long-term.^20–23^^,^^32^^,^^49^ Lastly, on average patients with relatively mild symptoms were included in this study, which limits generalizability to more severely ill patients. In acute patients, the impact of side effects on societal recovery and happiness might pale in comparison to the burden caused by the high disease burden.^23^

### Implications

Our results show that there is an association between subjective antipsychotic side-effect burden with societal functioning and happiness in a population of patients with a long-duration of illness (>17 years on average). This is in line with the experiences of antipsychotic users who point to the detrimental effects of side effects on their functioning and quality of life.^20–25^ Antipsychotic side effects can negatively impact self-esteem, motivation, (self)stigma, can instil feelings of hopelessness, and can lead to social withdrawal, restlessness, and depression.^20^^,^^23^^,^^28^^,^^29^^,^^65^^,^^66^ A shift away from a singular focus on clinical recovery to a more holistic (personal) recovery-oriented mental healthcare system has gained increased attention in both research and practice.^73^ To implement more recovery-oriented care, prescribers should be aware of and discuss the potential negative effects of antipsychotics in the long-term on societal recovery and happiness. Dose reduction might be especially prudent in patients with a relatively high dose who indicate that side effects hinder their societal recovery and overall well-being. A meta-analysis of RCTs showed that relapse prevention can be done with a relatively low dose in a population in long-term care.^19^ A recent open label randomized trial showed that a guided dose intervention, in which dose reduction was done slowly and was based on shared decision-making resulted in no more relapse compared to maintenance and improved quality of life compared to baseline.^74^ Still, the higher risk of relapse in the first 2 years after discontinuation or dose reduction seen in most RCTs^70^^,^^75^ needs to be taken into account when evaluating antipsychotic treatment.

In the analyses, we did not take into account which antipsychotic was used. Based on the different side-effect profiles and receptor affinities, the impact of antipsychotic treatment on societal recovery and happiness might vary strongly. Special attention needs to be afforded to patients with physical anticholinergic side effects who use antipsychotics with a strong affinity for muscarinic receptors such as quetiapine, olanzapine, and clozapine. These side effects might be a sign of cognitive/sedative anticholinergic side effects which sometimes can be hard to distinguish from negative symptoms.^47^

## Conclusion

The results of this study show a negative association of subjective side effects with societal recovery and happiness and a negative association of antipsychotic dose and antipsychotic polypharmacy with societal recovery in patients in long-term care for a psychotic disorder. Future research should focus on whether dose reductions or switching antipsychotics to account for side effects is beneficial for societal recovery and happiness in the long term.

## Appendix 1: Individual Association of 24 Antipsychotic Subjective Side Effects With Societal Recovery and Happiness

**Table A1:** Association of 24 Individual Subjective Antipsychotic Side Effect With *Societal Recovery*, Using Mixed-Effect Linear Regression Models. Coefficient, Standard Error, Z-Score, and *P*-Value for Mild Side Effects and Severe Side Effects With No Side Effect as the Reference Value

	**Mild side effect**		**Severe side effect**		
**Side effect**	**Coefficient (SE)**	**Z-score (*P*-value)**	**Coefficient (SE)**	**Z-score (*P*-value)**	**Side-effect group**
Slower reaction	**−0.112 (0.042)**	**−2.68 (.007)**	**−0.408 (0.072)**	**−5.69 (<.001)**	Cognition
Diminished creativity	**−**0.029 (0.046)	**−**0.64 (.523)	**−0.353 (0.068)**	**−5.21 (<.001)**	Cognition
Muscle stiffness	**−0.171 (0.047)**	**−3.67 (<.001)**	**−0.341 (0.072)**	**−4.71 (<.001)**	Extrapyramidal
Less energy for social interactions	**−0.106 (0.043)**	**−2.46 (.014)**	**−0.303 (0.068)**	**−4.46 (<.001)**	Mood
Difficulty remembering	**−**0.027 (0.043)	**−**0.64 (.525)	**−0.284 (0.065)**	**−4.39 (<.001)**	Cognition
Depression	**−0.118 (0.049)**	**−2.39 (.017)**	**−0.408 (0.094)**	**−4.36 (<.001)**	Mood
Constipation	**−0.201 (0.053)**	**−3.81 (<.001)**	**0.341 (0.079)**	**−4.30 (<.001)**	Anticholinergic
Blurry vision	**−**0.035 (0.048)	**−**0.73 (.468)	**−0.380 (0.094)**	**−4.04 (<.001)**	Anticholinergic
Physically tired	**−0.107 (0.042)**	**−2.54 (.011)**	**−0.223 (0.056)**	**−4.01 (<.001)**	Sedation
Dry mouth	**−0.141 (0.045)**	**−3.11 (.002)**	**−0.229 (0.064)**	**−3.56 (<.001)**	Anticholinergic
Harder to focus	**−0.141 (0.044)**	**−3.22 (.001)**	**−0.232 (0.072)**	**−3.22 (.001)**	Cognition
Urine loss	**−0.177 (0.054)**	**−3.27 (.001)**	**−0.288 (0.096)**	**−3.01 (.003)**	Cholinergic
Dizziness	**−**0.058 (0.047)	**−**1.24 (.214)	**−0.253 (0.086)**	**−2.93 (.003)**	Other
Blunted affect	**−**0.021 (0.043)	**−**0.48 (.634)	**−0.168 (0.065)**	**−2.58 (.010)**	Mood
Difficulties sitting still	**−0.118 (0.049)**	**−2.41 (.016)**	**−0.214 (0.086)**	**−2.48 (.013)**	Extrapyramidal
Tremors	**−0.126 (0.051)**	**−2.49 (.013)**	**−0.200 (0.091)**	**−2.18 (.029)**	Extrapyramidal
Lower libido	0.024 (0.048)	0.50 (.618)	**−0.127 (0.062)**	**−2.06 (.039)**	Sexual
Excessive salivation	**−0.095 (0.048)**	**−1.97 (.049)**	**−0.123 (0.062)**	**−1.98 (.048)**	Cholinergic
Difficulty reaching orgasm	0.077 (0.054)	1.44 (.151)	**−**0.103 (0.065)	**−**1.57 (.115)	Sexual
Difficulty waking up	0.012 (0.045)	0.26 (.791)	**−**0.066 (0.056)	**−**1.19 (.235)	Sedation/mood
Menstruating less frequently	0.069 (0.134)	0.51 (.609)	**−**0.168 (0.135)	**−**1.25 (.213)	Other
Increased appetite	0.030 (0.044)	0.68 (.496)	**−**0.019 (0.060)	**−**0.31 (.757)	Metabolism
Weight gain	0.058 (0.045)	1.28 (.200)	0.005 (0.054)	0.09 (.925)	Metabolism
Nausea	**−0.157 (0.062)**	**−2.54 (.011)**	**−**0.000 (0.116)	**−**0.00 (1.000)	Other

P-values ≤0.05 are statistically significant

**Table A2:** Association of 24 Individual Subjective Antipsychotic Side Effect with *Happiness*, Using Mixed-Effect Linear Regression Models. Coefficient, Standard Error, Z-Score and *P*-Value for Mild Side Effects and Severe Side Effects With No Side Effect as the Reference Value

	**Mild side effect**		**Severe side effect**		
**Side effect**	**Coefficient (SE)**	**Z-score (*P*-value)**	**Coefficient (SE)**	**Z-score (*P*-value)**	**Side-effect group**
Depression	**−0.611 (0.053)**	**−11.52 (<.001)**	**−1.70 (0.100)**	**−17.01 (<.001)**	Mood
Less energy for social interactions	**−0.457 (0.047)**	**−9.79 (<.001)**	**−1.068 (0.072)**	**−14.80 (<.001)**	Mood
Harder to focus	**−0.353 (0.047)**	**−7.45 (<.001)**	**−0.969 (0.077)**	**−12.58 (<.001)**	Cognition
Diminished creativity	**−0.401 (0.049)**	**−8.16 (<.001)**	**−0.869 (0.073)**	**−11.93 (<.001)**	Cognition
Difficulty remembering	**−0.265 (0.047)**	**−5.66 (<.001)**	**−0.738 (0.069)**	**−10.62 (<.001)**	Cognition
Physically tired	**−0.229 (0.047)**	**−0.489 (.001)**	**−0.620 (0.060)**	**−10.31 (<.001)**	Sedation
Blunted affect	**−0.344 (0.047)**	**−7.37 (<.001)**	**−0.693 (0.070)**	**−9.96 (<.001)**	Mood
Muscle stiffness	**−0.291 (0.050)**	**−5.79 (<.001)**	**−0.711 (0.078)**	**−9.13 (<.001)**	Extrapyramidal
Slower reaction	**−0.258 (0.046)**	**−5.66 (<.001)**	**−0.700 (0.077)**	**−9.11 (<.001)**	Cognition
Lower libido	**−0.254 (0.052)**	**−4.88 (<.001)**	**−0.594 (0.066)**	**−8.99 (<.001)**	Sexual
Difficulty waking up	**−0.147 (0.0490**	**−0.300 (.003)**	**−0.509 (0.059)**	**−8.58 (<.001)**	Sedation
Blurry vision	**−0.326 (0.051)**	**−6.33 (<.001)**	**−0.641 (0.099)**	**−6.50 (<.001)**	Anticholinergic
Dizziness	**−0.286 (0.051)**	**−5.64 (<.001)**	**−0.605 (0.093)**	**−6.49 (<.001)**	Other
Difficulty reaching orgasm	**−0.223 (0.059)**	**−3.75 (<.001)**	**−0.438 (0.070)**	**−6.21 (<.001)**	Sexual
Tremors	**−**0.101 (0.055)	**−**1.83 (.068)	**−0.512 (0.097)**	**−5.29 (<.001)**	Extrapyramidal
Dry mouth	**−0.213 (0.049)**	**−4.33 (<.001)**	**−0.353 (0.069)**	**−5.08 (<.001)**	Anticholinergic
Difficulties sitting still	**−0.238 (0.053)**	**−4.47 (<.001)**	**−0.466 (0.093)**	**−5.03 (<.001)**	Extrapyramidal
Constipation	**−0.123 (0.058)**	**−2.11 (.035)**	**−0.384 (0.086)**	**−4.46 (<.001)**	Anticholinergic
Excessive salivation	**−**0.006 (0.052)	**−**0.11 (.910)	**−0.246 (0.068)**	**−3.61 (<.001)**	Cholinergic
Nausea	**−0.253 (0.067)**	**−3.77 (<.001)**	**−0.422 (0.128)**	**−3.29 (.001)**	Other
Urine loss	**−0.135 (0.059)**	**−2.29 (.022)**	**−0.305 (0.103)**	**−2.96 (.003)**	Cholinergic
Increased appetite	**−**0.066 (0.087)	**−**0.077 (.433)	**−0.296 (0.106)**	**−2.78 (.005)**	Metabolism
Weight gain	**−**0.019 (0.049)	**−**0.39 (.699)	**−0.115 (0.057)**	**−2.01 (.045)**	Metabolism
Menstruating less frequently	0.0100 (0.152)	0.07 (.947)	**−**0.198 (0.148)	**−**1.34 (.180)	Hormonal

P-values ≤0.05 are statistically significant

**Table A3:** Association of 24 Individual Subjective Antipsychotic Side Effect With *Societal Recovery*, Using Mixed-Effect Linear Regression Models. Coefficient, Standard Error, Z-Score and *P*-Value for Mild Side Effects and Severe Side Effects With No Side Effect as the Reference Value Split Based on Sex

	**Men**				**Women**			
	**Mild side effect**		**Severe side effect**		**Mild side effect**		**Severe side effect**	
**Side effect**	**Coefficient (SE)**	**Z-score (*P*-value)**	**Coefficient (SE)**	**Z-score (*P*-value)**	**Coefficient (SE)**	**Z-score (*P*-value)**	**Coefficient (SE)**	**Z-score (*P*-value)**
Slower reaction	**−0.157 (0.052)**	**−3.03 (.002)**	**−0.442 (0.092)**	**−4.83 (<.001)**	**−**0.065 (0.069)	**−**0.94 (.349)	**−0.404 (0.114)**	**−3.54 (<.001)**
Diminished creativity	**−**0.004 (0.057)	**−**0.08 (.939)	**−0.417 (0.87)**	**−4.79 (<.001)**	**−**0.104 (0.076)	**−**1.36 (.174)	**−0.331 (0.107)**	**−3.10 (.002)**
Constipation	**−0.288 (0.068)**	**−4.21 (<.001)**	**−0.527 (0.118)**	**−4.47 (<.001)**	**−**0.121 (0.083)	**−**1.47 (.142)	**−0.264 (0.107)**	**−2.48 (.013)**
Physically tired	**−0.115 (0.051)**	**−2.24 (.025)**	**−0.314 (0.072)**	**−4.38 (<.001)**	**−**0.121 (0.072)	**−**1.67 (.096)	**−**0.148 (0.088)	**−**1.67 (.094)
Depression	**−0.157 (0.062)**	**−2.55 (.011)**	**−0.524 (0.128)**	**−4.11 (<.001)**	**−**0.053 (0.081)	**−**0.65 (.516)	**−0.307 (0.134)**	**−2.28 (.023)**
Blurry vision	**−**0.012 (0.060)	**−**0.21 (.836)	**−0.530 (0.129)**	**−4.10 (<.001)**	**−**0.115 (0.078)	**−**1.48 (.139)	**−0.321 (0.135)**	**−2.38 (.017)**
Less energy for social interactions	**−0.117 (0.053)**	**−2.20 (.028)**	**−0.326 (0.087)**	**−3.73 (<.001)**	**−**0.107 (0.073)	**−**1.47 (.143)	**−0.309 (0.106)**	**−2.91 (.004)**
Muscle stiffness	**−0.195 (0.058)**	**−3.37 (.001)**	**−0.349 (0.096)**	**−3.64 (<.001)**	**−0.171 (0.078)**	**−2.19 (.028)**	**−0.392 (0.109)**	**−3.59 (<.001)**
Harder to focus	**−0.182 (0.055)**	**−3.31 (.001)**	**−0.327 (0.095)**	**−3.45 (.001)**	**−**0.106 (0.071)	**−**1.50 (.133)	**−**0.163 (0.109)	**−**1.50 (.134)
Difficulty remembering	**−**0.091 (0.053)	**−**1.71 (.087)	**−0.290 (0.084)**	**−3.44 (.001)**	0.052 (0.071)	0.74 (.457)	**−0.333 (0.100)**	**−3.33 (.001)**
Blunted affect	**−**0.030 (0.053)	**−**0.57 (.570)	**−0.285 (0.083)**	**−3.44 (.001)**	**−**0.017 (0.074	**−**0.22 (.824)	**−**0.007 (0.104)	**−**0.06 (.950)
Lower libido	**−**0.087 (0.058)	**−**1.50 (.134)	**−0.258 (0.079)**	**−3.25 (.001)**	**0.271 (0.083)**	**3.28 (.001)**	0.064 (0.096)	0.67 (.506)
Difficulty reaching orgasm	0.039 (0.065)	0.61 (.542)	**−2.37 (0.080)**	**−2.95 (.003)**	0.200 (0.096)	2.07 (.038)	0.182 (0.111)	1.65 (.099)
Difficulties sitting still	**−**0.116 (0.061)	**−**1.90 (.058)	**−0.331 (0.111)**	**−2.99 (.003)**	**−**0.140 (0.082)	**−**1.70 (.089)	**−**0.047 (0.135)	**−**0.35 (.729)
Dizziness	**−0.150 (0.058)**	**−2.57 (.010)**	**−0.301 (0.116)**	**−2.61 (.009)**	0.075 (0.77)	0.98 (.326)	**−**0.220 (0.127)	**−**1.73 (.084)
Dry mouth	**−0.174 (0.56)**	**−3.08 (.002)**	**−0.213 (0.85)**	**−2.52 (.012)**	**−**0.133 (0.076)	**−**1.76 (.079)	**−0.325 (0.098)**	**−3.33 (.001)**
Tremors	**−0.157 (0.063)**	**−2.49 (.013)**	**−0.261 (0.123)**	**−2.12 (.034)**	**−**0.107 (0.083)	**−**1.28 (.200)	0.179 (0.133)	**−**1.34 (.182)
Urine loss	**−0.213 (0.070)**	**−3.04 (.002)**	**−0.269 (1.36)**	**−1.98 (.047)**	**−0.185 (0.084)**	**−2.20 (.028)**	**−0.439 (0.133)**	**−3.29 (.001)**
Increased appetite	0.022 (0.054)	0.42 (.676)	**−**0.119 (0.079)	**−**1.51 (.131)	0.035 (0.074)	0.47 (.637)	0.078 (0.092)	0.86 (.391)
Weight gain	0.021 (0.055)	0.38 (.707)	**−**0.085 (0.69)	**−**1.23 (.217)	0.131 (0.78)	1.69 (.091)	0.103 (0.084)	1.22 (.221)
Difficulty waking up	**−**0.010 (0.055)	**−**0.19 (.850)	**−**0.082 (0.071)	**−**1.16 (.245)	0.053 (0.078)	0.68 (.496)	**−**0.058 (0.090)	**−**0.64 (.523)
Excessive salivation	**−**0.106 (0.058)	**−**1.82 (.069)	**−**0.084 (0.077)	**−**1.09 (.276)	**−**0.044 (0.084)	**−**0.52 (.600)	**−**0.197 (0.105)	**−**1.89 (.059)
Nausea	**−**0.137 (0.80)	**−**1.72 (.086)	**−**0.078 (0.173)	**−**0.45 (.652)	**−**0.226 (0.096)	**−**2.35 (.019)	**−**0.038 (0.155)	**−**0.25 (.805)

P-values ≤0.05 are statistically significant

**Table A4:** Association of 24 Individual Subjective Antipsychotic Side Effect With *Happiness*, Using Mixed-Effect Linear Regression Models. Coefficient, Standard Error, Z-Score and *P*-Value for Mild Side Effects and Severe Side Effects With No Side Effect as the Reference Value Split Based on Sex

	**Men**				**Women**			
	**Mild side effect**		**Severe side effect**		**Mild side effect**		**Severe side effect**	
**Side effect**	**Coefficient (SE)**	**Z-score (*P*-value)**	**Coefficient (SE)**	**Z-score (*P*-value)**	**Coefficient (SE)**	**Z-score (*P*-value)**	**Coefficient (SE)**	**Z-score (*P*-value)**
Depression	**−0.507 (0.065)**	**−7.84 (<.001)**	**−1.60 (0.130)**	**−12.3 (<.001)**	**−0.790 (0.093)**	**−8.51 (<.001)**	**−1.84 (0.157)**	**−11.7 (<.001)**
Less energy for social interactions	**−0.383 (0.056)**	**−6.79 (<.001)**	**−1.03 (0.090)**	**−11.4 (<.001)**	**−0.591 (0.083)**	**−7.12 (<.001)**	**−1.14 (0.121)**	**−9.40 (<.001)**
Harder to focus	**−0.348 (0.058)**	**−5.99 (<.001)**	**−0.871 (0.098)**	**−8.86 (<.001)**	**−0.373 (0.082)**	**−4.54 (<.001)**	**−1.11 (0.125)**	**−8.90 (<.001)**
Diminished creativity	**−0.356 (0.059)**	**−5.99 (<.001)**	**0.774 (0.091)**	**−8.48 (<.001)**	**−0.483 (0.087)**	**−5.53 (<.001)**	**−1.02 (0.121)**	**−8.38 (<.001)**
Lower libido	**−0.314 (0.061)**	**−5.14 (<.001)**	**−0.653 (0.082)**	**−7.99 (<.001)**	**−**0.103 (0.098)	**−**1.05 (.292)	**−0.484 (0.113)**	**−4.30 (<.001)**
Muscle stiffness	**−0.219 (0.061)**	**−3.58 (<.001)**	**−0.758 (0.101)**	**−7.53 (<.001)**	**−0.434 (0.089)**	**−4.88 (<.001)**	**−0.670 (0.125)**	**−5.38 (<.001)**
Slower reaction	**−0.238 (0.055)**	**−4.32 (<.001)**	**−0.688 (0.095)**	**−7.28 (<.001)**	**−0.297 (0.081)**	**−3.67 (<.001)**	**−0.723 (0.133)**	**−5.45 (<.001)**
Physically tired	**−0.205 (0.056)**	**−3.66 (<.001)**	**−0.537 (0.075)**	**−7.21 (<.001)**	**−0.293 (0.085)**	**−3.43 (.001)**	**−0.762 (0.103)**	**−7.39 (<.001)**
Difficulty remembering	**−0.254 (0.056)**	**−4.50 (<.001)**	**−0.625 (0.088)**	**−7.11 (<.001)**	**−0.307 (0.084)**	**−3.67 (<.001)**	**−0.925 (0.115)**	**−8.04 (<.001)**
Dizziness	**−0.307 (0.062)**	**−4.95 (<.001)**	**−0.731 (0.119)**	**−6.23 (<.001)**	**−0.244 (0.089)**	**−2.75 (.006)**	**−0.414 (0.151)**	**−2.74 (.006)**
Blunted affect	**−0.248 (0.056)**	**−4.44 (<.001)**	**−0.509 (0.086)**	**−5.90 (<.001)**	**−0.529 (0.084)**	**−6.29 (<.001)**	**−1.01 (0.118)**	**−8.56 (<.001)**
Difficulty reaching orgasm	**−0.214 (0.068)**	**−3.14 (.002)**	**−0.430 (0.084)**	**−5.13 (<.001)**	**−0.234 (0.119)**	**−1.98 (.048)**	**−0.438 (0.128)**	**−3.43 (.001)**
Blurry vision	**−0.327 (0.063)**	**−5.21 (<.001)**	**−0.651 (0.128)**	**−5.09 (<.001)**	**−0.328 (0.090)**	**−3.65 (<.001)**	**−0.607 (0.157)**	**−3.86 (<.001)**
Difficulties sitting still	**−0.267 (0.064)**	**−4.17 (<.001)**	**−0.564 (0.115)**	**−4.92 (<.001)**	**−**0.175 (0.096)	**−**1.83 (.068)	**−**0.294 (0.157)	**−**1.87 (.062)
Tremors	**−0.087 (0.067)**	**−1.29 (.197)**	**−0.512 (0.125)**	**−4.11 (<.001)**	**−**0.120 (0.096)	**−**1.25 (.213)	**−0.508 (0.155)**	**−3.27 (.001)**
Dry mouth	**−0.203 (0.060)**	**−3.41 (.001)**	**−0.360 (0.088)**	**−4.08 (<.001)**	**−0.225 (0.087)**	**−2.57 (.010)**	**−0.345 (0.115)**	**−3.01 (.003)**
Constipation	**−**0.136 (0.073)	**−**1.87 (.061)	**−0.381 (0.121)**	**3.15 (.002)**	**−**0.119 (0.098)	**−**1.22 (.222)	**−0.394 (0.127)**	**−3.09 (.002)**
Difficulty waking up	**−0.130 (0.059)**	**−2.21 (.027)**	**−0.491 (0.191)**	**−2.57 (.010)**	**−0.188 (0.088)**	**−2.14 (.032)**	**−0.587 (0.102)**	**−5.74 (<.001)**
Excessive salivation	0.009 (0.062)	0.14 (.891)	**−0.173 (0.083)**	**−2.09 (.037)**	**−**0.044 (0.096)	**−**0.46 (.647)	**−0.402 (0.120)**	**−3.36 (.001)**
Urine loss	**−0.163 (0.075)**	**−2.16 (.031)**	**−**0.250 (0.144)	**−**1.74 (.081)	**−**0.104 (0.096)	**−**1.09 (.275)	**−0.366 (0.153)**	**−2.40 (.017)**
Nausea	**−0.260 (0.084)**	**−3.09 (.002)**	**−**0.316 (0.177)	**−**1.78 (.075)	**−0.256 (0.112)**	**−2.29 (.022)**	**−0.491 (0.191)**	**−2.57 (.010)**
Increased appetite	**0.114 (0.057)**	**2.00 (.045)**	0.116 (0.082)	1.42 (.155)	**−**0.066 (0.087)	**−**0.77 (.443)	**−0.296 (0.106)**	**−2.78 (.005)**
Weight gain	**−**0.004 (0.058)	**−**0.06 (.948)	**−**0.041 (0.071)	**−**0.58 (.565)	**−**0.064 (0.091)	**−**0.70 (.486)	**−0.247 (0.098)**	**−2.54 (.011)**

P-values ≤0.05 are statistically significant
